# High Throughput Screening for Small Molecule Enhancers of the Interferon Signaling Pathway to Drive Next-Generation Antiviral Drug Discovery

**DOI:** 10.1371/journal.pone.0036594

**Published:** 2012-05-04

**Authors:** Dhara A. Patel, Anand C. Patel, William C. Nolan, Yong Zhang, Michael J. Holtzman

**Affiliations:** 1 Drug Discovery Program, Pulmonary and Critical Care Medicine, Department of Medicine, Washington University School of Medicine, Saint Louis, Missouri, United States of America; 2 Department of Pediatrics, Washington University School of Medicine, Saint Louis, Missouri, United States of America; 3 Department of Cell Biology, Washington University School of Medicine, Saint Louis, Missouri, United States of America; Lady Davis Institute for Medical Research, Canada

## Abstract

Most of current strategies for antiviral therapeutics target the virus specifically and directly, but an alternative approach to drug discovery might be to enhance the immune response to a broad range of viruses. Based on clinical observation in humans and successful genetic strategies in experimental models, we reasoned that an improved interferon (IFN) signaling system might better protect against viral infection. Here we aimed to identify small molecular weight compounds that might mimic this beneficial effect and improve antiviral defense. Accordingly, we developed a cell-based high-throughput screening (HTS) assay to identify small molecules that enhance the IFN signaling pathway components. The assay is based on a phenotypic screen for increased IFN-stimulated response element (ISRE) activity in a fully automated and robust format (Z′>0.7). Application of this assay system to a library of 2240 compounds (including 2160 already approved or approvable drugs) led to the identification of 64 compounds with significant ISRE activity. From these, we chose the anthracycline antibiotic, idarubicin, for further validation and mechanism based on activity in the sub-µM range. We found that idarubicin action to increase ISRE activity was manifest by other members of this drug class and was independent of cytotoxic or topoisomerase inhibitory effects as well as endogenous IFN signaling or production. We also observed that this compound conferred a consequent increase in IFN-stimulated gene (ISG) expression and a significant antiviral effect using a similar dose-range in a cell-culture system inoculated with encephalomyocarditis virus (EMCV). The antiviral effect was also found at compound concentrations below the ones observed for cytotoxicity. Taken together, our results provide proof of concept for using activators of components of the IFN signaling pathway to improve IFN efficacy and antiviral immune defense as well as a validated HTS approach to identify small molecules that might achieve this therapeutic benefit.

## Introduction

**Figure 1:**
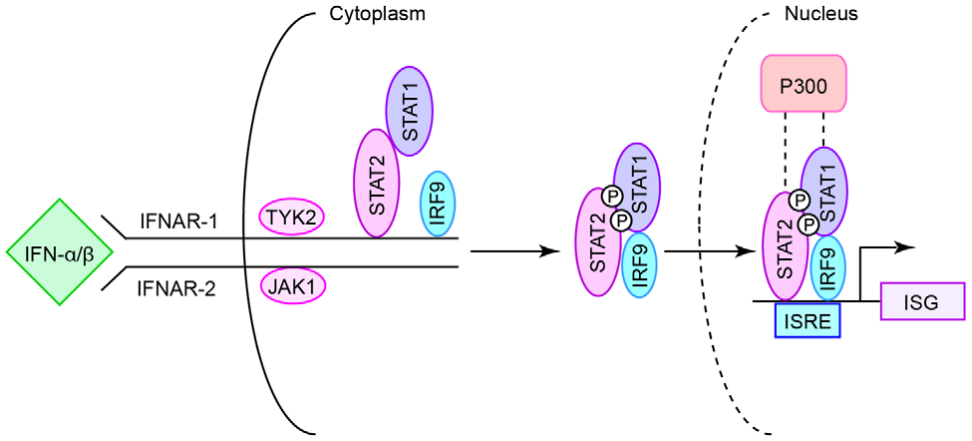
Scheme for IFN signal transduction. Type I IFN signaling starts by activation of the IFN-α/β receptor (IFNAR) and subsequent activation of the IFNAR1-associated TYK2 and IFNAR2-associated JAK1, with consequent recruitment of STAT2. Phosphorylation of STAT2 enables reruitment of STAT1 and release of the phosphorylated STAT1-STAT2 heterodimer bound to IRF-9. This complex binds to the IFN stimulated response element (ISRE) and in concert with recruited transcriptional co-activators such as p300/CBP then drives IFN-stimulated gene (ISG) transcription.

There has been significant progress in the development of vaccines and therapeutics against viruses, but there are still major gaps in medical therapy for some of the most common types of viral infections. For these types of infections, vaccines can still be ineffective due to new and emergent strains and can exhibit significant off-target effects [1], [2]. Similarly, the efficacy of antiviral therapeutics can often be limited by pathogen resistance as another sign of the difficulty in keeping up with rapidly evolving viral genomes [3]–[9]. An alternative to agents that specifically and directly target the virus itself is the possibility of improving natural host defense against a broad range of viruses. Although antiviral defense exhibits significant complexity and redundancy, one system that stands out as a useful target for improvement is the one based on the action of interferons (IFNs). And within this IFN system, which is similarly complex, the STAT1 transcription factor is remarkable as a central component that is critical for the functional activity of each type of IFN (Figure 1). Consequently, genetic loss of STAT1 function causes a marked susceptibility to viral infection in mice and humans [10]–[12]. Moreover, modification of STAT1 to a form) that improves the efficiency of IFN signal transduction can result in improved control of viral infection [13]. These observations indicate that the IFN-signaling pathway is subject to a so-called “rheo-STAT” adjustment wherein down-regulation causes increased susceptibility to viral infection whereas up-regulation might lead to increased efficiencies for IFN-stimulated gene (ISG) expression and control of infection [14].

In the present study, we aimed to mimic the beneficial actions of STAT1 modification with a small molecule that also enhances the activity of the IFN signaling pathway. We describe here the development of a high-throughput screening (HTS) system for novel small molecular weight compounds (so-called “small molecules”) that might increase ISG expression and antiviral activity. To develop this screening system, we generated cell lines that stably express the human interferon-stimulated response element (ISRE) driving a luciferase reporter gene. The ISRE gene promoter element is responsible for type I IFN signaling that mediates host defense against a wide range of viruses [15], [16]. After establishing that the ISRE-reporter cell line responded linearly to IFN-β concentration and treatment time, we converted the assay to an automated format for a screen against already approved or approvable drugs. We also screened a library of phosphatase inhibitors that might mediate increased STAT1 phosphorylation-activation. Our analysis identified a series of diverse compounds capable of significantly increasing ISRE activity. One compound in particular, the anthracycline antibiotic idarubicin hydrochloride, was used to explore mechanism of action and to validate the proposal that small molecules can enhance ISRE activity to drive higher levels of ISG expression and improved control of viral level. The findings provide for the concept that current antiviral therapeutics act directly and specifically on viral proteins whereas next-generation antivirals might act to enhance host immunity against a broad range of viruses. Either alone or together, these approaches might better address the current need for more effective treatment against common as well as new and emergent viral infections.

## Results

### Generation of a cell-based assay system for ISRE activity

**Figure 2:**
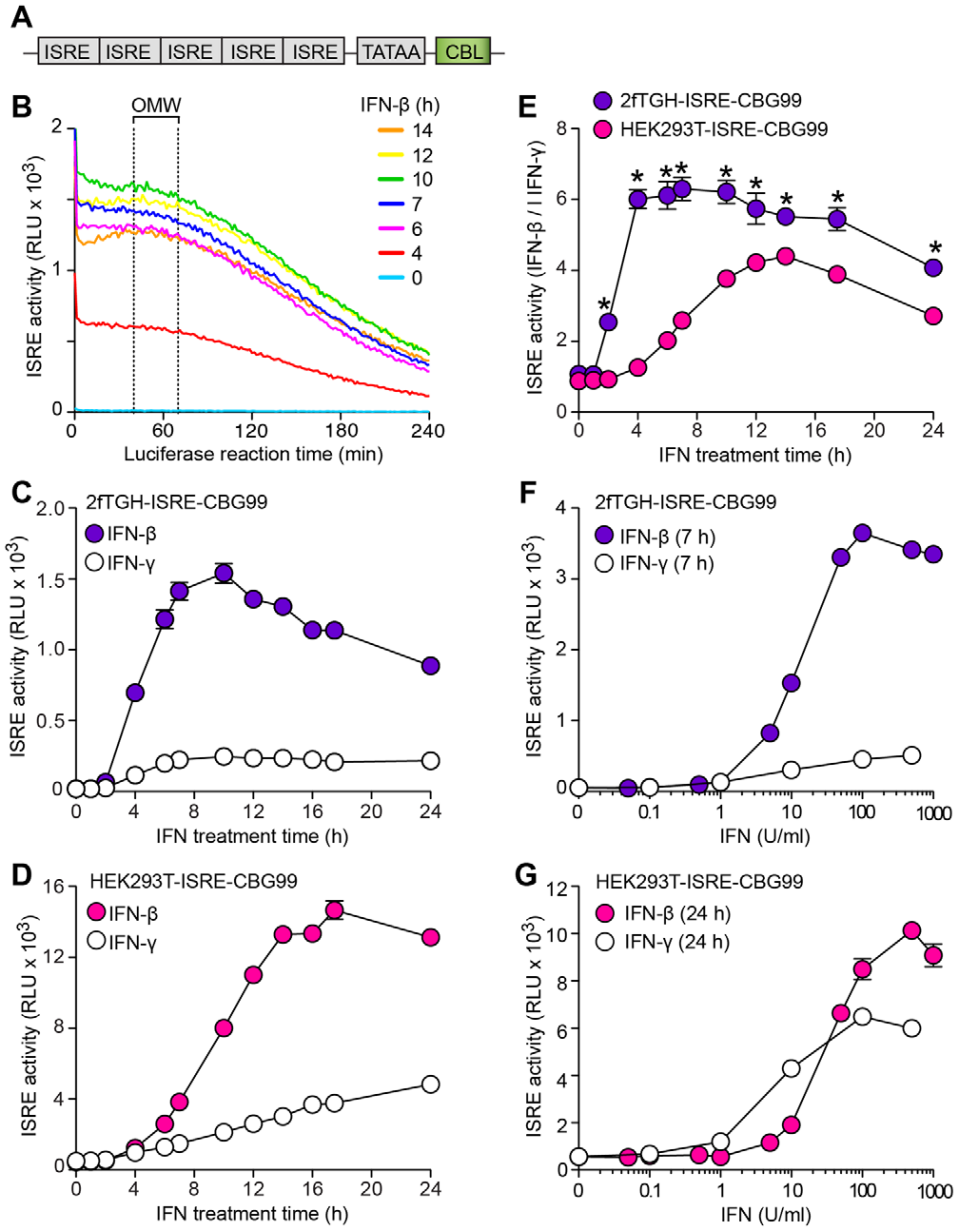
IFN responsivness of the ISRE-promoter luciferase-reporter system. (A) Schematic representation of the vector construct (ISRE-CBG99) used to establish stable cell lines for monitoring ISRE activity driving a click beetle luciferase (CBL) reporter gene. (B) 2fTGH cells stably expressing the ISRE-CBG99 construct (2fTGH-ISRE-CBG99 cells) were treated with IFN-β (1000 U/ml for 0–14 h) and then monitored for luciferase-catalyzed luminescence over 0–4 h. Signal maximum was found at 50 min, and the optimal measurement window (OMW) with at least 90% preservation of signal) was found at 40–70 min. (C) Time course for effect of IFN-β (1000 U/ml) and IFN-γ (100 U/ml) on ISRE activity in 2fTGH-ISRE-CBG99 cells. (D) Corresponding time course for HEK293T-ISRE-CBG99 cells. (E) Ratio of ISRE activities when each cell line is treated with saturating concentrations of IFN-β versus IFN-γ for 0–24 h. * indicates significant differences between values for 2fTGH versus HEK293T cell lines. (F) Concentration-response curves for effect of IFN-β and IFN-γ on ISRE activity in 2fTGH-ISRE-CBG99 cells. (G) Corresponding concentration-response for HEK293T-ISRE-CBG99 cells.

Based on the observation that STAT1-CC-expressing cells show increased activity of the endogenous ISRE promoter element [13], we established cell lines that stably expressed an ISRE-containing gene promoter driving a click beetle luciferase reporter gene CBG99luc (Figure 2A). We used 2fTGH cells (the parental line for STAT1-deficient U3A cells) as well as HEK293T cells that both express endogenous STAT1. Clonal lines showing IFN-β-inducible ISRE-promoter driven luciferase activity were designated 2fTGH- or HEK293T-ISRE-CBG99 cells. For initial assay development, we used the 2fTGH- and HEK293T-ISRE-CBG99 cells to establish an optimal luciferase light reaction time for both cell-lines. Based on luminescence signal stability over the time course of the reaction, an optimal readout window of 40–70 min after the start of the reaction was chosen for subsequent experiments (Figure 2B). After optimization of cell growth time, response to various IFN-β treatment times and concentrations were tested in 2fTGH and HEK293T cell lines. Each cell line exhibited a distinct IFN-β treatment time for maximal signal: 7–12 h for 2fTGH-ISRE-CBG99 cells and 14–24 h for HEK293T-ISRE-CBG99 cells. Although a lower signal magnitude was obtained with 2fTGH-ISRE-CBG99 cells compared to HEK293T-ISRE-CBG99 cells, the 2fTGH-ISRE-CBG99 cells show more specificity for IFN-β (compared to IFN-γ) treatment at all IFN-β treatment time periods tested (Figure 2E) and over a range of IFN-β (and IFN-γ) concentrations (Figure 2F, G). Thus, the 2fTGH-ISRE-CBG99 cells were chosen for further assay development.

### Assay automation and miniaturization

To achieve assay automation and miniaturization, the ISRE activity assay was first automated in 96-well plates and then reformatted for 384-well plates. In the 384-well format, the assay exhibited a near-maximal signal at 8000 cells per well and consistent well-to-well and plate-to-plate reproducibility (Figure S1). In 96- and 384-well formats, signal to background (S/B) ratios, coefficients of variance, and Z′-factors achieved excellent performance in comparison to published standards [17], [18]. Representative results for 2fTGH-ISRE-CBG99 cells treated with IFN-β (1000 U/ml) for 7 h compared to 1% DMSO vehicle alone are provided in Table 1. The results indicate the development of a quantitative and specific cell-based HTS assay of IFN-responsive gene promoter activity.

**Table 1:** Table **1.** Well-to-well reproducibility of automated ISRE activity assay for 2fTGH-ISRE-CBG99 cells treated with IFN-β (1000 U/ml for 7 h) versus DMSO vehicle alone.

Format	S/B	CV (%)	Z′-factor
96 well	206	5.1	0.84
384 well	112	10.9	0.70

Abbreviations: S/B, signal to background ratio; CV, coefficient of variation.

### Cell-based HTS of a small-molecule library

**Figure 3:**
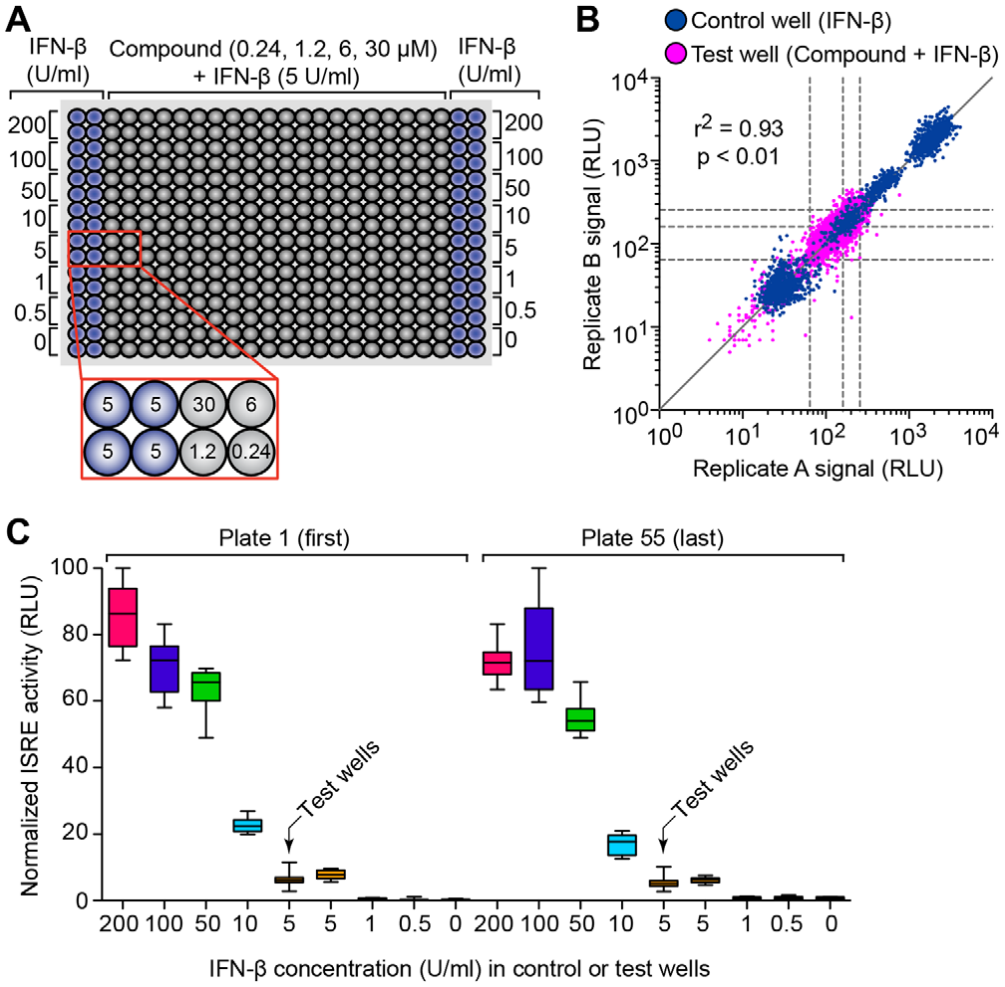
Reproducible and stable signals from HTS of the chemical library. (A) Schematic of assay plate map used in screening the chemical compound library. (B) Reproducibility of raw data between duplicate assay plates for control wells containing IFN-β (0–200 U/ml) and test wells containing compound plus IFN-β (5 U/ml). Dashed lines indicate median ± SD of each data set from the test wells. (C) Signal consistency through a full screen of 56 assay plates. Values represent replicate A of assay plate 1 (the first plate) and plate 55 (the last plate). Box plots represent median and 25^th^ and 75^th^ percentile, and whiskers indicate minimum and maximum of data. Arrows indicate values from test wells.

We used the automated ISRE-activity assay to perform a screen of a 2240 chemical compound library. This library consisted of 2160 compounds from the Johns Hopkins Clinical Compound Library (JHCCL) of FDA approved or approvable drugs [19], [20]. In addition, we included 33 compounds from the Screen-Well Phosphatase Inhibitor Library based on the observation that the improvement in IFN signaling in STAT1-CC-expressing cells correlated with prolonged phosphorylation of STAT1 and STAT2 [13]. Each compound was tested at 4 different concentrations (0.24, 1.2, 6 and 30 μM) and simultaneous treatment with IFN-β at 5 U/ml, the concentration at the initial inflexion of the IFN concentration-response curve (as shown in Figure 2F). These treatment conditions were duplicated on a second plate. Each assay plate also contained control wells containing a range of IFN-β concentrations (0–200 U/ml) in quadruplicate (Figure 3A). This arrangement achieved excellent signal reproducibility between duplicate compound plates as well as signal consistency through the full screening run of 56 assay plates (28 duplicate pairs) (Figure 3B, C).

**Figure 4:**
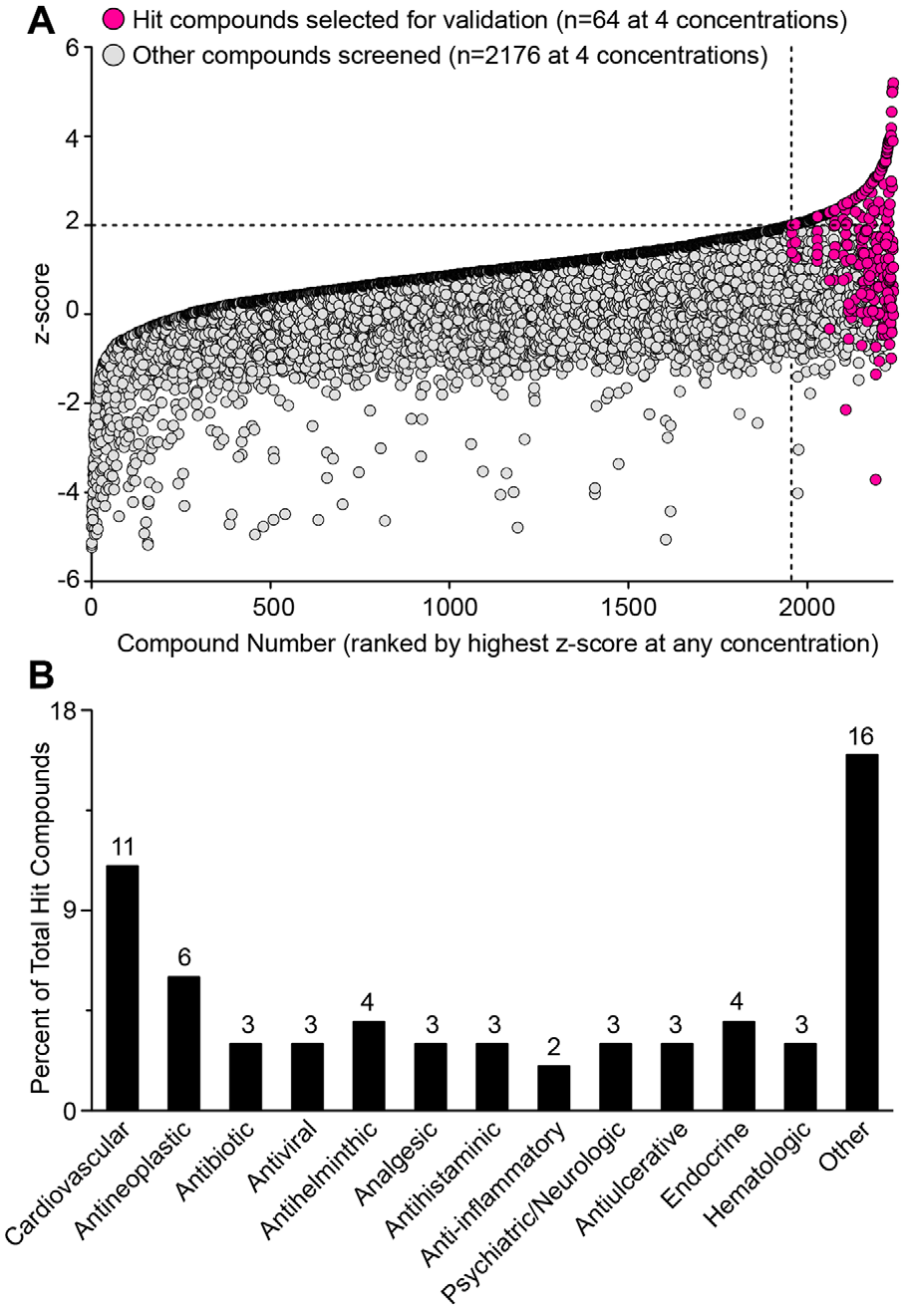
Hit selection and therapeutic class of drugs from primary HTS assay for ISRE activation. (A) Plot of z-scores for each of the 2240 compounds subjected to the primary screen for ISRE activation. Values represent mean of two replicates per compound concentration. Compounds are ranked by highest z-score achieved at any of the four compound concentrations tested. Dashed lines demarcate the compounds with z-scores greater than 2 SD above the mean; and red color denotes the 64 hit compounds selected from this group for validation. (B) The distribution of the 64 hit compounds into various therapeutic classes. Values indicate the number of compounds in each class.

After raw data were normalized, scaled to z-scores, and summarized, we found that 321 data points (out of a total of 8960 data points representing the 2240 compounds tested at 4 concentrations) had an ISRE activity z-score ≥2 (Figure 4A). This data set represented 285 individual compounds, as some compounds had an ISRE activity z-score ≥2 at more than one dose. Of these 285 compounds, 64 hit compounds (2.9% of the total compound library) were selected for validation based on a combination of dose-response characteristics and inter-replicate reproducibility. This approach captured all 20 of the compounds with the highest z-scores. In support of re-purposing as a drug discovery strategy, the 64 screening hits were found in a broad range of drug classes (Figure 4B).

### Screening hit idarubicin increases ISRE activity independent of IFN

**Figure 5:**
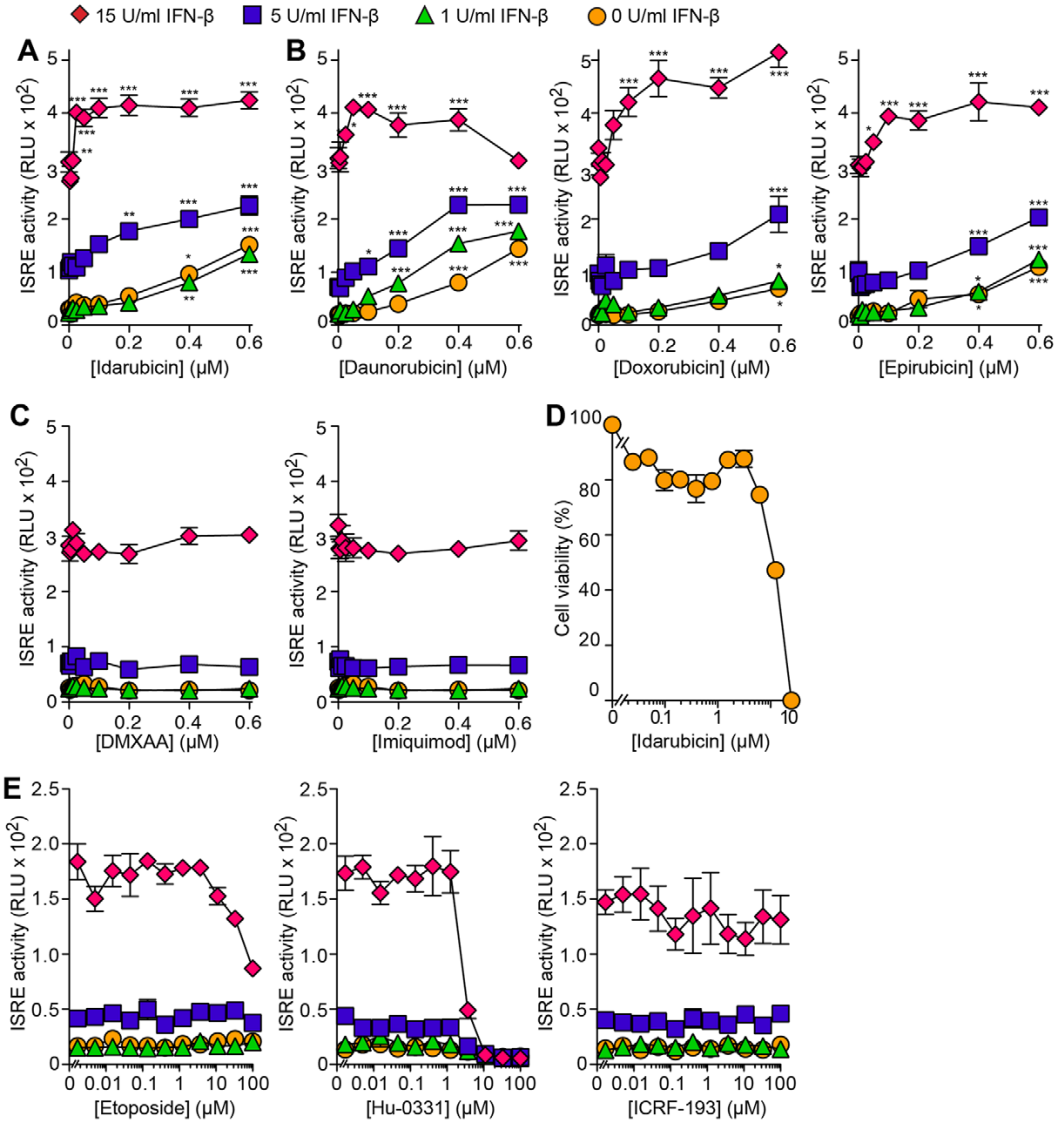
Validation and specifcity of idarubicin capacity for ISRE activation. (A) Idarubicin concentration-response for ISRE activity without and with treatment with IFN-β (1, 5, and 15 U/ml). 2fTGH-ISRE-CBG99 cells were treated with idarubicin and IFN-β for 8 h. Overall significance for idarubicin dose P<0.0001, IFN-β dose p<0.0001, and Interaction p<0.0001. The p-values for individual comparisons (versus control without idarubicin) are from Bonferroni post-tests from two-way ANOVA (idarubicin x IFN dose). Symbols: * p<0.05, ** p<0.01, *** p<0.001. (B) Corresponding data for daunorubicin, doxorubicin, and epirubicin concentration-response for ISRE activity. (C) Corresponding data for DMXAA and Imiquimod concentration-response for ISRE activity. Overall significance for compound dose p = ns, IFN-β dose p<0.0001, interaction p = ns. (D) Idarubicin toxicity determined using Alamar Blue metabolism assay. Cells were treated with drug for 12 h. (E) Etoposide, Hu-0331, and ICRF-193 (0–100 µM) concentration-response for ISRE activity without and with IFN-β (1, 5, and15 U/ml). Overall significance for ICRF-193 dose p = ns, IFN-β dose p<0.0001, interaction p = ns; for Etoposide and HU-0331 dose, there is a significant inhibitory effect on ISRE activity, p<0.0001, IFN-β dose p<0.0001, interaction p<0.0001.

Each of the 64 primary hits was subjected to primary validation for ISRE activity over a broader range of concentrations of drug and IFN-β (0–15 U/ml). Among the primary and confirmed screening hits, idarubicin hydrochloride ranked highest in potency for enhancing ISRE activity (i.e., idarubicin exhibited a significant effect at a lower concentration than other compounds). During the ISRE validation, we found that idarubicin caused a concentration-dependent increase in ISRE activity over a range of IFN-β treatment concentrations, with highly significant effects as low as 25 nM idarubicin in combination with 15 U/ml IFN-β (Figure 5A).

The structure for idarubicin shows characteristic features of an anthracycline antibiotic unrelated to any other antiviral compound in clinical use. To further validate the effect of idarubicin on ISRE activity, we tested three other anthracyclines (daunorubicin, doxorubicin, epirubicin) with very similar chemical structures to idarubicin. Each of these compounds also showed a capacity to significantly increase ISRE activity (Figure 5B). In addition, we found that the immune activators DMXAA (Vadimezan) and Imiquimod did not cause any increase in ISRE activity in the same concentration range (Figure 5C). These compounds appear to directly activate immune cells (including increased IFN production) [21], [22]. However, for the present work, we specifically studied non-hematopoietic cells since that population appears critical for STAT1-mediated defense against at least some types of viruses [11].

We also found a cytotoxic effect of idarubicin that is consistent with previous observations [23], [24]. Under the present conditions, the major effect of idarubicin on cell viability was detected at drug concentrations >3 µM, so that the ISRE-activating effect of idarubicin occurred at concentrations below those that cause a major effect on cell viability (Figure 5D). Nonetheless, idarubicin is best known as an anti-neoplastic agent that acts via DNA intercalation and topoisomerase II inhibition [25]. To determine whether the effect of idarubicin on ISRE activity is related to topoisomerase II inhibition, we tested three other potent topoisomerase inhibitors Etoposide, Hu-0331, and ICRF-193 up to concentrations known to cause topoisomerase II inhibition [26]–[28]. In contrast to idarubicin, these other compounds caused no significant increase in ISRE activity (Figure 5E). In fact, two of the topoisomerase inhibitors (Etoposide and Hu-0331) caused a decrease in ISRE activity in concert with cytotoxic effects at higher concentrations. These findings indicate that the capacity of idarubicin to activate the ISRE component of the IFN signaling pathway occurs independently of topoisomerase inhibition. Together, the findings provide evidence of idarubicin capacity to increase ISRE activity independent of the anti-neoplastic properties of the drug.

**Figure 6:**
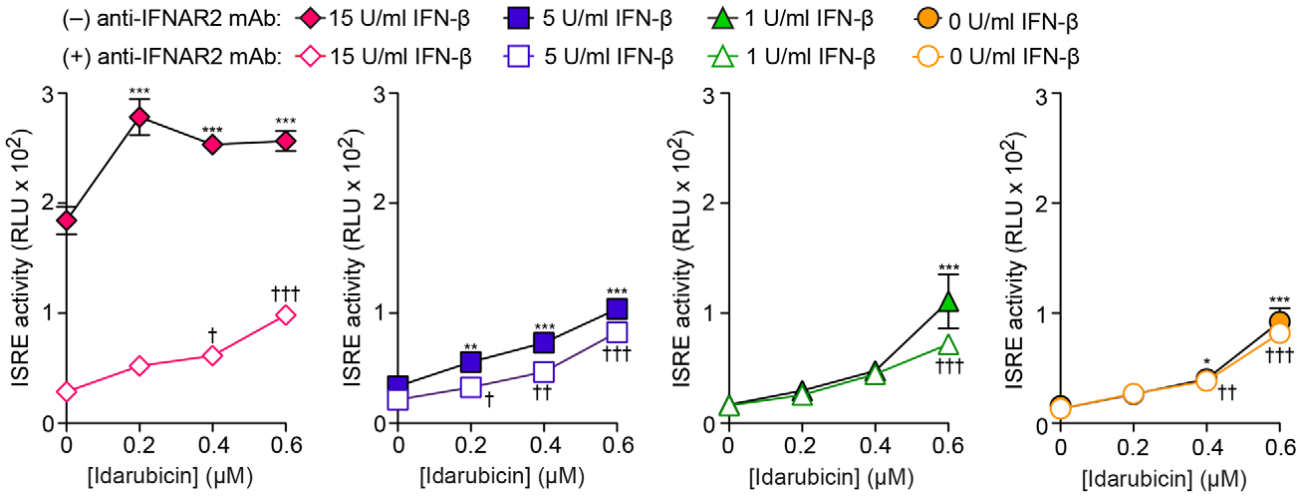
Effect of IFN-receptor blockade on idarubicin stimulation of ISRE activity. Idarubicin concentration-response for ISRE activity without and with treatment with IFN-β (1, 5, and15 U/ml) in the absence or presence of anti-IFNAR2 blocking mAb. *,† = p<0.05 ; **,†† = p<0.01; ***,††† = p<0.001. p-values for individual comparisons are from Bonferroni post-tests from repeated measures two-way ANOVA, comparing the 0 nM Idarubicin condition to the other drug doses. For 15 U/ml IFN-β, overall significance: anti-IFNARII, p<0.0001, IDA dose, p<0.0001; interaction, p = 0.0023. For 5 U/ml IFN-β, overall significance: anti-IFNARII, p<0.0001, IDA dose, p<0.0001; interaction, p = 0.3294. For 1 U/ml IFN-β, overall significance: anti-IFNARII, p<0.0892, IDA dose, p<0.0001; interaction, p = 0.1542. For 0 U/ml IFN-β, overall significance: anti-IFNARII, p<0.4432, IDA dose, p<0.0001; interaction, p = 0.7677.

We also observed that the effect of idarubin and the other anthracyclines on ISRE activity occurred at a lower concentration of drug when IFN-β was co-administered, particularly at the highest concentration of IFN-β (15 U/ml) (Figure 5A). For example, the EC_50_ for idarubicin decreased as the concentration of IFN-β increased (Table 2). These findings suggested that idarubicin might somehow interact with the IFN signaling pathway. In that regard, we also found that the effect of idarubicin on ISRE activity was observed under baseline conditions when there was no detectable production of endogenous IFN-β and no administration of exogenous IFN-β (data not shown and Figure 5A). These results suggested that the effect of idarubicin on ISRE activity is independent of IFN production or action. Indeed, we also found that the effect of idarubicin on ISRE activity persisted without change during effective IFN-α/β receptor 2 (IFNAR2) blockade (Figure 6). Together, the findings indicate that idarubicin causes an increase in ISRE activity independent of IFN production or IFN–IFN-receptor interaction and instead acts downstream of ligand-receptor binding in the IFN signaling pathway.

**Table 2:** Idarubicin effect on ISRE activity relative to IFN-β treatment concentration.

IFN-β (U/ml)	EC_50_ *	Confidence Interval	R^2^
0	394.7	378.7–411.2	0.93
1	361.9	321.1–408.0	0.92
5	224.7	149.2–338.5	0.69
15	15.97	11.75–21.70	0.68

* Values for EC_50_ were calculated by fitting the data to a four-parameter concentration-response curve for idarubicin effect on ISRE activity as described in Methods.

### Idarubicin enhances IFN-driven ISG expression and antiviral activity

**Figure 7:**
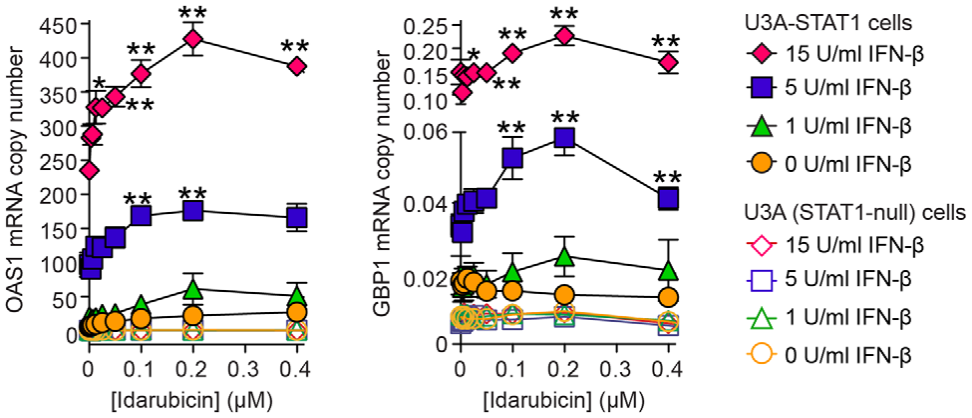
Idarubicin induces STAT1-dependent gene expression. U3A-STAT1 and U3A (STAT1-null) cells were treated with idarubicin for 1.5 h followed by IFN-β for 12 h and then determination of *OAS1* and *GBP1* mRNA levels using real-time quantitative PCR assay. Overall significance for idarubicin dose p<0.0001, IFN-β dose p<0.0001, and interaction p<0.0001. The p-values for individual comparisons (vs 0 idarubicin control) are from Bonferroni post-tests from two-way ANOVA (idarubicin x IFN dose). Symbols: * p<0.01, ** p<0.001.

We subjected idarubicin to further validation as an ISRE activator in assays of ISG expression and antiviral activity. For ISG expression, U3A (STAT1-null) and U3A-STAT1 cells were treated with a range of concentrations of idarubicin and IFN-β and then harvested for gene expression using quantitative real-time PCR assay. We found that idarubicin increased the expression of the antiviral gene 2′,5′-oligoadenylate synthetase 1 (*OAS1*), particularly with IFN-β treatment (Figure 7). There was no effect of drug on ISG expression in STAT1-null U3A cells, indicating that the drug is specific for STAT1-dependent gene expression. We found similar results for the antiviral ISG guanylate-binding protein 1 (*GBP1*) and three other ISGs (*MX1*, *PARP9*, and *IRF1*) (Figure 6 and data not shown).

**Figure 8:**
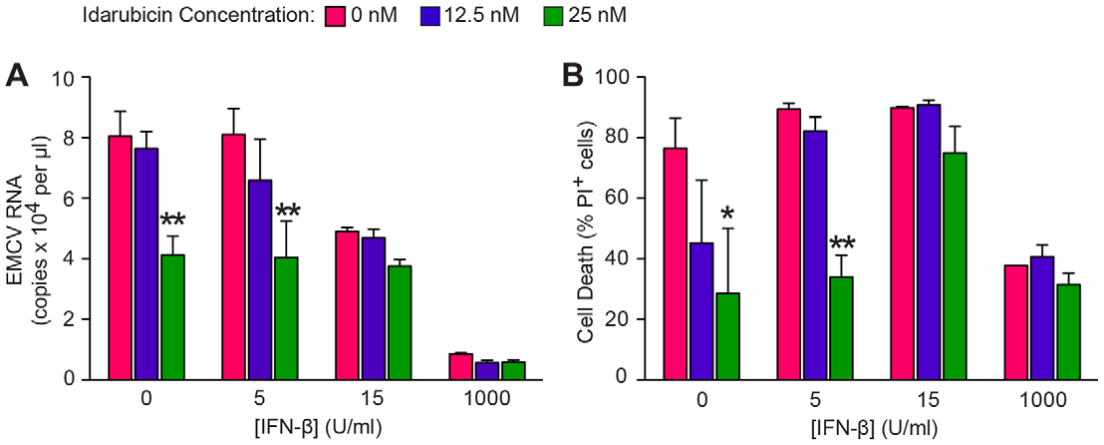
Idarubicin provides control of viral titer. (A) 2fTGH cells were treated with idarubicin or DMSO vehicle and with IFN-β for 6 h and then inoculated with EMCV (MOI 1) for 1 h. Viral titers in cell supernatant were measured at 28 h after inoculation using real-time quantitative PCR assay for EMCV RNA. Values represent mean ± SE (n = 3 biological replicates, n = 2 technical replicates). Overall significance for idarubicin dose p<0.0001, IFN dose p<0.0001, and Interaction p<0.05. (B) For the protocol used in (A), the corresponding levels of virus-induced cell toxicity based on cell viability from propidium iodide (PI) staining. Overall significance for idarubicin dose P<0.0001, IFN dose p<0.0001, and Interaction p = ns. The p-values for individual comparisons (vs 0 idarubicin control) are from Bonferroni post-tests from two-way ANOVA (idarubicin x IFN dose). Symbols: *p<0.05, **p<0.01, ***p<0.001.

For antiviral activity, 2fTGH cells were treated with idarubicin along with or without IFN-β and then assessed for control of encephalomyocarditis virus (EMCV) levels and virus-induced cytopathic effect. We selected EMCV since it was previously found to be sensitive to STAT1-CC-dependent improvement in IFN signaling [13]. In the present experiments, we found that idarubicin treatment (at a relatively low concentration of 25 nM) caused a significant decrease in EMCV titer at baseline and with IFN-β treatment (at a relatively low concentration of 5 U/ml) (Figure 8A). In addition, we observed that idarubicin-dependent improvement in viral control translates into a significant decrease in viral cytopathic effect under those same treatment conditions (Figure 8B). Higher concentrations of idarubicin in combination with IFN-β treatment caused a significant cytotoxic effect (data not shown), consistent with the antineoplastic properties of the drug. Nonetheless, the results with a relatively low concentration of idarubicin provide proof-of-concept that a small molecule activator of the ISRE component of the IFN signaling pathway will allow for increased ISG expression and improved control of viral level.

## Discussion

The present study was undertaken to discover antiviral therapeutics that broadly increase host defense. We focused on the IFN system that is central to the antiviral response, although we recognized that other labs have pursued this target with limited success in the past. Some of these previous investigators have used administration of IFN itself to increase the antiviral response, but for this therapeutic goal and others, the side effects of IFN administration have proven to be rate-limiting [29]. Similarly, other investigators have attempted to boost IFN production, e.g., through administration Toll-like receptor (TLR) agonists CpG or Imiquimod, but these agents have also caused similar side effects [30]–[33]. The small molecule DMXAA activates multiple immune pathways (NF-κB, TBK1/IRF3, NOD, and MAP kinase) but was ineffective as an antiviral unless it was administered before infection [34]–[37]. To circumvent at least some of these issues with IFN production, toxicity, and specificity, we therefore pursued the goal of antiviral drug discovery with a novel screening approach for identifying small molecule enhancers that might selectively boost the activity or efficiency of the IFN signaling pathway.

Our specific approach was based on previous success with the use of a modified STAT1 signaling pathway. In this work, we demonstrated that a designer form of STAT1 (designated STAT1-CC based on double-cysteine substitutions) was able to enhance IFN signaling and better control viral replication [13]. Although STAT1-CC gene expression would be challenging to translate to practical application, the mechanism of action served as a guide to design a screening strategy to identify small molecules that could mimic the antiviral benefit. In that regard, we also recognized that phenotypic screening approaches have proven to be more effective than target-based approaches for the discovery of first-in-class small molecule drugs [38]. Thus, target-based approaches (defined as direct drug action on a particular target) allow for analysis and refinement of structure and function but can also waste resources when the molecular hypotheses used to design screening assays may not be relevant to the disease. Meanwhile, phenotypic screening can take longer in terms of hit-to-lead development but provide more proteins in the pathway to be targeted and do not require prior knowledge of molecular mechanisms of action. Most importantly, the activity found in phenotypic-based approaches is often more effectively translated into therapeutic impact in disease models. For the present work, we take advantage of both of these strategies to some extent and devise a screen that incorporates molecular mechanism (i.e., enhancing a specific type of IFN signaling pathway) and the need to achieve phenotype (i.e., identifying any compound that could increase this type of signaling pathway regardless of specific mechanism).

Considering these factors and observations in the STAT1-CC model system, we designed a cell-based luciferase reporter assay for measuring type I-dependent ISRE activity. This assay proved to yield excellent signal-to-background and Ź factors, specificity for IFN-β treatment over IFN-γ treatment, and suitability for automation and screening. Furthermore, because the construct design uses the Click Beetle Green luciferase, the assay can be paired with other luciferase reporter genes to develop dual color assays to report activity of other signaling pathways, including the type II IFN-γ activated sequence (GAS) promoter activity that mediates defense against intracellular bacteria. A related approach was used to screen for small molecules that increase GAS activity for anti-proliferative and pro-apoptotic effects in cancer cells [39]. Others screened for compounds that inhibit Type I IFN production and signaling [40]. An ISRE-RFP reporter system has also been used to screen for assessing the effects of immuno-stimulatory RNA [41]. Others have used a less directed approach to screen for compounds that might use any mechanism to decrease viral levels [40], [42]–[45]. However, to our knowledge, the present study conducts the first semi-quantitative screen measuring ISRE activity to discover small molecule enhancers of the type I IFN signaling pathway as broad-spectrum antiviral therapeutics.

Our primary screen identified idarubicin on the basis of its capacity to significantly increase ISRE activity. Subsequent validation assays demonstrated that idarubicin facilitates STAT1-dependent ISG expression and STAT1-directed control of viral replication and cytopathic effect. While others previously reported the antiviral properties of anthracyclines some time ago, no mechanism of their antiviral action was elucidated [46]–[48]. In the present study, we observed drug-induced cytotoxicity in a dose-range similar to those reported previously [23], [24], however, we establish that the effect of idarubicin on the antiviral IFN pathway is independent of cytotoxicity and topoisomerase inhibition. Because idarubicin enhances the IFN signaling pathway output, we questioned whether the drug might also cause IFN-driven cell death. However, we found no increase in cytotoxicity in cells treated with idarubicin and IFN together compared to cells treated with idarubicin alone. We also found that the idarubicin concentrations for activating the ISRE component of the IFN signaling pathway were significantly less than those required for major cytotoxicity. Thus, we conclude that idarubicin effect on IFN signaling is distinct from the effect on DNA-based cytotoxicity. The dose dependency of these effects underlines the need to conduct screening at multiple concentrations of test compounds, particularly lower concentrations that prevent false negative hits due to cytotoxic effects.

In a further analysis of drug mechanism, our study demonstrates that the antiviral activity of idarubicin and other closely related anthracyclines is derived from enhancing the activity of the type I IFN signaling pathway. Our data further show that the enhancing effect of idarubicin is based on ISRE activation and ISG expression independent of IFN production or IFN–IFN-receptor interaction, since the effect of idarubicin is unchanged by IFN-receptor blockade. These findings suggest that idarubicin activation of the ISRE is due to an action in the IFN signaling pathway distal to ligand-receptor binding, e.g., at the level of receptor-associated JAK kinases or further downstream at the formation, transport, binding, and/or assembly of the ISRE transcriptional complex. In that regard, anthracyclines are known to inhibit DNA and RNA synthesis by intercalating between base pairs of the DNA/RNA strand to prevent replication, but whether this mechanism can affect ISRE or other gene promoter elements still needs to be defined. The present screening approach overcomes the uncertainty in molecular mechanism by using a phenotypic (rather than a target-based) screening approach and thereby captures compounds that increase the activity of the IFN signaling pathway by either established or undefined mechanisms.

In sum, we describe and validate a phenotypic screening strategy to identify small molecules that enhance the activity of the type I IFN signaling pathway and consequently improve antiviral host defense. This approach is designed to lead to discovery of drugs with activity against a broad range of viruses for clinical application as well as experimental tool compounds to further understand IFN-dependent immune mechanisms. Current approaches to defining the basis for IFN signal transduction, particularly in vivo, often rely on complex transgenic and gene targeting approaches. Thus, the use of small molecule enhancers (SMEs) of the IFN signaling pathway may provide much greater flexibility and ease of application to achieve transient adjustment of IFN-related actions and consequent scientific and clinical benefit. Our approach should thereby prove useful to discover drugs with activity against a broad range of viruses as well as effectiveness in other conditions (e.g., multiple sclerosis and melanoma) where the efficacy of IFN treatment might benefit from enhancing the IFN signaling pathway.

## Materials and Methods

### Stimulating agents and chemical compounds

IFN-β and IFN-γ were obtained from PBL Interferon source (Piscataway, NJ), diluted and aliquoted according of manufacturer's recommendation, and stored at −80°C. The Johns Hopkins Clinical Compound Library (JHCCL) was obtained from Dr. David Sullivan at the Johns Hopkins University [19], [20]. The Screen-Well Phosphatase Inhibitor Library was obtained from Enzo Life Sciences (Farmingdale, NY). All other chemical compounds were obtained from Sigma Aldrich (St. Louis, MO).

### Vector construction and stable transduction of cell lines

To construct the pISRE-CBG99 vector, we first generated a 5x-repeat of the ISRE sequence and a TATAA box (5xISRE-TATAA) in the pUCMinusMCS vector from Blue Heron Biotechnology (Bothell, WA) and then cloned this sequence into the Chroma-Luc pCBG99-Basic reporter vector from Promega (Madison, WI) and ligated into Xma1 and Nco1 sites using T4 DNA ligase from Life Technologies (Carlsbad, CA). The DNA sequence of the resultant pISRE-CBG99 vector was confirmed by carrying out BigDye Terminator v3.1 sequencing reactions (Life Technologies) on an ABI capillary sequencer. This vector and the pPUR selection vector from Clontech (Mountain View, CA) were co-transfected at a 9∶1 ratio into 2fTGH or HEK293T cells to increase the likelihood that cells tolerating puromycin selection (0.5 μg/ml) contained one or more copies of pISRE-CBG99 in addition to pPUR. The 2fTGH cells [49] were obtained from G. Stark (Cleveland Clinic), and HEK293T cells [50], [51] were obtained from T. Brett (Washington University). Transfection was performed using Fugene6 transfection reagent from Roche Applied Science (Indianapolis, IN). Limiting dilution was used to obtain individual cell clones that were then screened for luciferase-mediated luminescence after treatment with IFN-β (1000 U/ml) on a BioTek Synergy 4 multimode plate reader (BioTek, Winooski, VT). Clonal cells exhibiting stable expression were then used for further assay development.

### Optimization and validation of ISRE activity-luciferase reporter assay

To optimize the luciferase light reaction, we tested a series of flash and glow luminescent substrate systems in both lysed and live 2fTGH-ISRE-CBG99 and HEK293T-ISRE-CBG99 cells, including D-luciferin (Fisher Scientific, Pittsburgh, PA), the Chroma-Glo Luciferase assay system from Promega and the steadylite plus reporter gene assay system from Perkin Elmer (Waltham, MA) under a range of incubation conditions. The steadylite plus system was selected based on kinetic profile. Effects of IFN-β concentration and treatment time were assessed with all other variables constant.

### Assay automation

The assay was automated in a 96-well format with a customized and fully integrated robotic system. The system equipment included: a Caliper Sciclone ALH 3000 workstation (Perkin Elmer) and a EL406 washer (BioTek) for liquid handling, an automated Liconic incubator (Thermo Scientific) for cold storage of plates, an automated Cytomat incubator (Thermo Scientific) for cell culture environment, a separate hotel for storage of plates at room temperature, a Synergy 4 plate reader, a Flexiseal plate heat sealer (K Biosciences, Beverly, MA), a Caliper Twister II, and a Beckman Sagian Orca robotic arm on a linear rail (Beckman Coulter, Fullerton, CA). Construction allowed for transfer of plates, reagents, and plasticware between all instruments, so that there was no need for any manual interference during screening assays. This entire system was enclosed in a custom-made laminar flow hood to allow for HTS screening capability under BSL2 sterile conditions. After the system demonstrated satisfactory performance in a 96-well format, the assay was miniaturized to a 384-well format and re-tested for reproducibility and stability under IFN-β and vehicle (1% DMSO) treatment conditions.

### High-throughput screen

To achieve simultaneous treatment of cells with IFN-β and various compound concentrations and to avoid reagent degradation over time, the screen was run in a modular manner with a precise timeline (Figure S2). The first step included production of plates with appropriate concentrations of compound and IFN-β and then storage at 4°C. A separate plate was made for each of the four compound concentrations (0.24, 1.2, 6 and 30 μM). The Twister II, Sciclone, Orca, and Liconic cold storage incubator handled this step. For the second step, cells were plated at 8000 cells per well in 384-well assay plates (n = 56). This step was accomplished in seven batches (8 assay plates per batch) using the Sciclone. A uniform suspension of cells was maintained by intermittent mixing on the Sciclone deck between cell plating. Simultaneously, the compound stock plates were sealed using the Flexiseal and stacked back into a Twister II rack for storage. For the third step, cells were allowed to grow for 11 h and then were treated with compound and IFN solutions. This step required that a plate containing cells be brought from the Cytomat incubator to the Sciclone deck in concert with a set of compound/IFN dilutions plates from the cold storage incubator. Cell treatments were timed so that each assay plate would be incubated for 10.3 h before the final step of performing the luciferase assay. For this last step, robotics were programmed so that each assay plate developed the luciferase light reaction for 40 min at 25°C in the plate hotel and then was delivered to the Synergy 4 plate reader for determination of luminescence. For this assay, the BioTek EL406 washer was used for aspiration of media and dispersion of substrate. In entirety, the screen took 41.6 h to complete.

### HTS data analysis

The raw data from the HTS assay were subjected to statistical analysis using cellHTS2 [52], [54], a software package designed for the analysis of HTS data as part of the Bioconductor project for statistical computing [55]. Raw data were normalized using the plate median method [52], [56]. Next, a z-score transformation was applied to center and scale the data across the experiment. Replicates for a given compound at a given dose (N = 2 for each dose/compound combination) were then mean summarized. A z-score threshold of ≥2 was chosen to identify potential hits. Thereafter, to reach a smaller and tractable set of hits to validate experimentally, we took advantage of testing each compound at four concentrations. Specifically, we used self-organizing maps analysis to cluster hit compounds by shape of the dose-response curve. The significance of change from dose to dose (0.24 to 1.2, 1.2 to 6, and 6 to 30) was also analyzed using linear models and moderated F-statistics as implemented in the limma package [57] in Bioconductor [55]. The concentration-response curves for each compound were then visually inspected, using scatter plots generated in TIBCO Spotfire DecisionSite (TIBCO, Palo Alto, CA), with respect to the shape of the curve and reproducibility between replicates. Compounds showing an erratic concentration-response (e.g. increase, then decrease, and increase again in ISRE activity with increasing concentration) were rejected. Compounds with a consistent increase or decrease in response with increasing drug concentration or good efficacy at any concentration were included for further validation. This approach led to selection of 64 compounds for further validation, including compounds with the 20 highest z-scores.

### Hit validation, drug potency (EC_50_) estimation, and IFN dependence

Hits from the primary screen were validated using the ISRE activity-luciferase reporter assay over a broad range of compound concentrations (0.01–25 µM) in the absence or presence of IFN-β (1, 5, and 15 U/ml). To determine drug potency, as defined by half-maximal effective concentration (EC_50_), this data was fit to a four-parameter concentration-response curve as described previously [58] using the log agonist concentration versus response, variable slope algorithm in GraphPad Prism 5 software (La Jolla, CA) where Y = Bottom + (Top-Bottom)/(1+10̂((LogEC50-X)*HillSlope)). To determine whether compound effect depended on IFN production, the ISRE activity-luciferase reporter assay was also performed in the presence of mouse anti-human IFN-α/β receptor chain 2 (IFNAR2) blocking mAb (clone MMHAE-2; Millipore, Billerica, MA) at a concentration of 4 µg/ml.

### Compound toxicity assay using Alamar Blue

A resazurin (Alamar Blue) metabolism assay was used to assess cell toxicity during compound treatment [59]. For these experiments, cells were treated with compound or an equivalent concentration of vehicle (DMSO) for 12 h, and the medium was replaced with fresh medium containing resazurin (80% dilution of Tox-8 kit, Sigma-Aldrich, St. Louis, MO). After 1.5 h at 37°C under standard culture conditions, the fluorescence of the resultant product resorufin was measured using the Synergy 4 plate reader. Wells with cells containing no compound (DMSO alone) and wells containing no cells were used as 100% and 0% viability controls. Data were normalized to calculate percentage viability.

### Analysis of ISG expression

Expression of ISG's was assessed with real-time quantitative PCR assay for the corresponding mRNA level. For these experiments, U3A and U3A-STAT1 cells were first treated with the programmed combination of compound and IFN-β for 12 h and then were washed twice with cold Dulbecco's PBS followed by lysis with Cells-to-cDNA II lysis buffer (Life Technologies) and treatment with DNase according to the manufacturer's instructions. The U3A cells were obtained from G. Stark (Cleveland Clinic) and complemented with STAT1 to generate U3A-STAT1 cells as described previously [13]. A 25-μl aliquot of the cell lysate was used to generate cDNA using the High Capacity Reverse Transcription Kit (Life Technologies). The resulting cDNA was quantified using the Quant-iT OliGreen ssDNA kit (Life Technologies). Average cDNA concentration was 71±25 ng/μl. Subsequent PCR assays were performed in adherence with MIQE guidelines [60], [61], including the design of assays for ISGs (*OAS1* and *GBP1*) and normalizer gene ornithine decarboxylase antizyme (*OAZ1*). The normalizer gene *OAZ1* was selected and validated for cell samples treated with and without IFN-β. In brief, candidate normalizer genes were selected from a combination of invariant genes selected from previous microarray data [11], [62], [63] and prior large-scale analyses of publicly available microarray data [64], [65]. These candidates were then tested using real-time quantitative PCR assays. Comparison of candidate normalizer gene expression between various IFN treatment and infection conditions using multiple software packages [66 68] led to the selection of the OAZ1 as the normalizer gene. Primers and probes for real-time quantitative PCR assays were designed using the ProbeFinder design algorithm (Roche Applied Science). For OAS1, 5′-ggtggagttcgatgtgctg-3′ and 5′-aggtttatagccgccagtca-3′ were used as forward and reverse primers, along with UPL probe #37 (Roche Applied Science). A plasmid containing OAS1 transcript variant 2 cDNA (Ref ID NM_002534, Origene, Rockville, MD) was used as a standard for absolute quantitation of OAS1 copy number. For GBP1, 5′-ttccaaaactaaaactctttcagga-3′ and 5′-tgctgatggcattgacgtag-3′ were used as forward and reverse primers, along with UPL probe #85. A plasmid containing the GBP1 cDNA (Clone ID: 3606865, Thermo Open Biosystems, Huntsville, AL) was used as a standard. The IDT PrimeTime pre-designed assay Hs.PT.42.328511.g (Integrated DNA Technologies, Coralville, IA) was used for OAZ1. A cDNA vector was used for OAZ1 as well (Clone ID: LIFESEQ913650, Thermo Open Biosystems). Data were collected on a LightCycler 480 instrument (Roche Applied Science). Quantification cycle values were calculated using a second derivative maxima algorithm as implemented in the Lightcycler 480 software.

### Viral inoculation and assessment of cell viability

Cells were cultured overnight and then treated with compound and IFN-β for 6 h. Thereafter, cells were washed and then were inoculated with EMCV (strain VR-129B, ATCC, Manassas, VA) for 1 h at MOI 1 as described previously [13]. Cells were then washed twice and cultured in medium containing 2% fetal bovine serum for 28 h. At that time, cell supernatants were used to determine viral titer based on real-time quantitative PCR assay for EMCV RNA with 5′-ctgccttcggtgtcgc-3′ (forward primer), 5′-tgggtcgaatcaaagttggag-3′ (reverse primer), and 5′caaggttttgagcgtgtctacgatgtgg-3′ (probe). A TA plasmid containing the EMCV 3D protein was used as a standard for absolute quantitation of viral copy number. In addition, cell viability was determined using the Cellomics Arrayscan VTi high content imager (Thermo Scientific). For this assay, 15 images per well were obtained with a 10x objective. After background subtraction, cells were identified by nuclei stained by cell permeable dye Hoechst 33342. Propidium iodide fluorescence was quantified by defining a boundary of 2 pixels around the nuclei and then gating on a cell population that showed higher staining. For each sample replicate, cytotoxicity was calculated as the percentage of cells that showed increased propidium iodide staining based on samples of at least 5000 cells per well.

## Supporting Information

Figure S1 **Miniaturization and automation of the ISRE activity assay.** (A) Effect of cell density on ISRE activity as a function fo IFN-β concentration in 384-well plates. B) Well-to-well reproducibility for the assay performed in 384-well automated format using 8000 cells per well.
(EPS)

Figure S2 **Scheme for the HTS automation protocol.** Each box represents one of the sequential steps in the screening process. Timing and time allotment for each step is also indicated.
(EPS)
